# IL-10-Producing B Cells Regulate T Helper Cell Immune Responses during 1,3-β-Glucan-Induced Lung Inflammation

**DOI:** 10.3389/fimmu.2017.00414

**Published:** 2017-04-06

**Authors:** Fangwei Liu, Xiaowei Lu, Wujing Dai, Yiping Lu, Chao Li, Sitong Du, Ying Chen, Dong Weng, Jie Chen

**Affiliations:** ^1^Division of Pneumoconiosis, School of Public Health, China Medical University, Shenyang, China; ^2^Department of Respiratory Medicine, Shanghai Pulmonary Hospital, Tongji University School of Medicine, Shanghai, China

**Keywords:** regulatory B cells, T helper immune responses, 1,3-β-glucan, lung inflammation, occupational disease

## Abstract

With the rapid development of industry and farm, fungi contamination widely exists in occupational environment. Inhalation of fungi-contaminated organic dust results in hypersensitivity pneumonitis. 1,3-β-Glucan is a major cell wall component of fungus and is considered as a biomarker of fungi exposure. Current studies showed that 1,3-β-glucan exposure induced lung inflammation, which involved uncontrolled T helper (Th) cell immune responses, such as Th1, Th2, Th17, and regulatory T cell (Treg). A recently identified IL-10-producing B cells (B10) was reported in regulating immune homeostasis. However, its regulatory role in hypersensitivity pneumonitis is still subject to debate. In our study, we comprehensively investigated the role of B10 and the relationship between B10 and Treg in 1,3-β-glucan-induced lung inflammation. Mice with insufficient B10 exhibited more inflammatory cells accumulation and severer pathological inflammatory changes. Insufficient B10 led to increasing Th1, Th2, and Th17 responses and restricted Treg function. Depletion of Treg before the onset of inflammation could suppress B10. Whereas, Treg depletion only at the late stage of inflammation failed to affect B10. Our study demonstrated that insufficient B10 aggravated the lung inflammation mediated by dynamic shifts in Th immune responses after 1,3-β-glucan exposure. The regulatory function of B10 on Th immune responses might be associated with Treg and IL-10. Treg could only interact with B10 at an early stage.

## Introduction

Organic dust exists widely in the occupational environment, such as farm and industries that processing hay, paper, fur, or household waste (1, 2). Inhalation of organic dust induces acute or chronic lung inflammation, named as hypersensitivity pneumonitis. And its latent period ranges from several hours to several months (3). Although it is curable in some cases, granulomatous inflammation may result in irreversible fibrosis (4). The etiology of hypersensitivity pneumonitis in organic dust working environments includes kinds of microorganism and animal antigens. Epidemiologic study demonstrates that fungi concentration is much higher in jute, fur, or silk-processing workshops, which seriously damage the workers’ health in occupational environment. Organic dust exposure is often characterized by high levels of viable fungi and fungal spores, which drew considerable attention (5, 6). 1,3-β-Glucan is a major cell wall component of fungi and is considered as a biomarker of fungi exposure (7).

Existing evidence supports an obvious relationship between the airborne level of 1,3-β-glucan and the respiratory symptom (4). Abundant immune cells are reported to be involved in 1,3-β-glucan-induced lung inflammation, including neutrophils, macrophages, and lymphocytes especially. This suggests that both innate and adaptive immune responses took part in 1,3-β-glucan-induced lung inflammation. Patients with hypersensitivity pneumonitis exhibit high percentage of lymphocytes in peripheral blood, which indicates a crucial role of lymphocytes in the development of hypersensitivity pneumonitis (8). We have previously showed that multiple CD4^+^ T lymphocyte responses dominated in different stages after 1,3-β-glucan exposure, including T helper (Th)1, Th2, Th17, and regulatory T cell (Treg) (9, 10). Exogenous 1,3-β-glucan induces numerous kinds of inflammatory cytokines and chemokine through NF-kB and NLRP3 signal pathways (11, 12). And then activates the Th1 response and Th2 response in sequence. Th17 response also participates in the initial acute inflammation. Besides, we have previously demonstrated that Treg impacted on the Th1/Th2 immune responses skewed to Th1 predominance. Treg depletion modulates the process of 1,3-β-glucan-induced lung inflammation and the later fibrosis pathological change (9).

Besides these classical T cell subtypes, a novel regulatory B cell is reported to be capable of controlling autoimmune disease, allergic disease, and tumorigenesis (13–15). B cell depletion increases asthma-like airway inflammation in mice (16, 17). Activation of CD19^+^CD1d^hi^ B cells suppresses allergic lung inflammation (18–20). CD19^+^CD24^hi^CD38^hi^ B cells possess regulatory function in pneumonia patients and are associated with later development of its complication (21). Although there is various phenotypes for regulatory B cells, such as CD1d^hi^CD5^+^, CD21^+^CD23^+^, or TIM-1^+^, several reports describe an IL-10-producing B cells (B10) in controlling chronic intestinal inflammation and experimental autoimmune encephalomyelitis (22, 23). Therefore, CD19 and IL-10 are used as markers for B10 (24). B10 could modulate Th immune responses by affecting the secretion of inflammatory cytokines, such as IFN-γ, IL-12, and IL-17 (19, 25). Study *in vitro* demonstrates that IL-10-overexpressing B cells were able to suppress the secretion of inflammatory cytokines, the maturation of dendritic cells, and the antigen-specific proliferation (26). Transfer of antigen-specific IL-10-depleted splenic B cells restores experimental ovalbumin (OVA)-induced allergic airway inflammation (16). CD22 was dominantly expressed on B cells and considered to play an important role in regulating B cells by binding to its ligand. Preferential depletion of B10 by using anti-CD22 antibody could amplify the focal and systematic inflammation (27, 28). However, the regulatory mechanism of B10 in lung inflammation is still subject to debate. Some believe that IL-10 is instrumental for B10s suppressive effect (16, 29). And Treg is reported to help the regulatory function of B10 (20, 30). However, other evidence shows the regulatory role of B10 is Treg-independent (31, 32). Whether the regulatory function of B10 relies on Treg is still dubious. The regulatory mechanism of B10 in 1,3-β-glucan-induced lung inflammation is not well understood.

In this study, we investigated the role of B10 during the development of 1,3-β-glucan-induced lung inflammation. The regulatory effect of B10 on 1,3-β-glucan-induced Th responses was investigated, and the reciprocal relationship between B10 and Treg was discussed. We concluded that insufficient B10 aggravated the lung inflammation *via* promoting different Th immune responses during different stages after 1,3-β-glucan exposure. The regulatory function of B10 on Th immune responses might be associated with Treg and IL-10. Treg could only interact with B10 at an early stage.

## Animals and Methods

### Animals

Healthy female C57BL/6 mice at 6–8 weeks age were purchased from SLAC Laboratory Animal Co. Ltd. (Shanghai, China). All animals were housed in a specific-pathogen-free environment and maintained on standard mouse chow at an environmental temperature of 24 ± 1°C, with 12 h light/12 h dark cycles, and water *ad libitum*.

### 1,3-β-Glucan Exposure

Eighty-four female mice were randomly allocated into five groups according to weight as follows: saline group, saline + anti-CD22 group, glucan group, glucan + anti-CD22 group, and glucan + anti-CD25 group. Zymosan A (1,3-β-glucan) from *Saccharomyces cerevisiae* (Z4250), purchased from Sigma-Aldrich Inc. (St. Louis, MO, USA), was dissolved in sterile saline to a final concentration of 6 mg/ml. Female mice were anesthetized with intraperitoneal injection of 2% pentobarbital sodium (45 mg/kg body weight). Mice received 0.3 mg/50 μl zymosan solution intratracheally to induce lung inflammation. Control mice received 50-μl sterile saline at the same time.

### B10/Treg Depletion

To deplete CD19^+^IL10^+^ regulatory B cells, mice were injected intraperitoneally with 300 μg anti-CD22 antibody (KH2014176, F239, Sangon Biotech, Shanghai, China) 1 day before 1,3-β-glucan exposure and repeatedly treated every 7 days for continuing depletion (27, 28). To deplete CD4^+^Foxp3^+^ Treg, mice received intraperitoneal injection of 100 μg of anti-CD25 mAb (PC61; BioLegend, San Diego, CA, USA) as described previously (9). IgG1 was used as control.

### Bronchoalveolar Lavage

**Figure 1:**
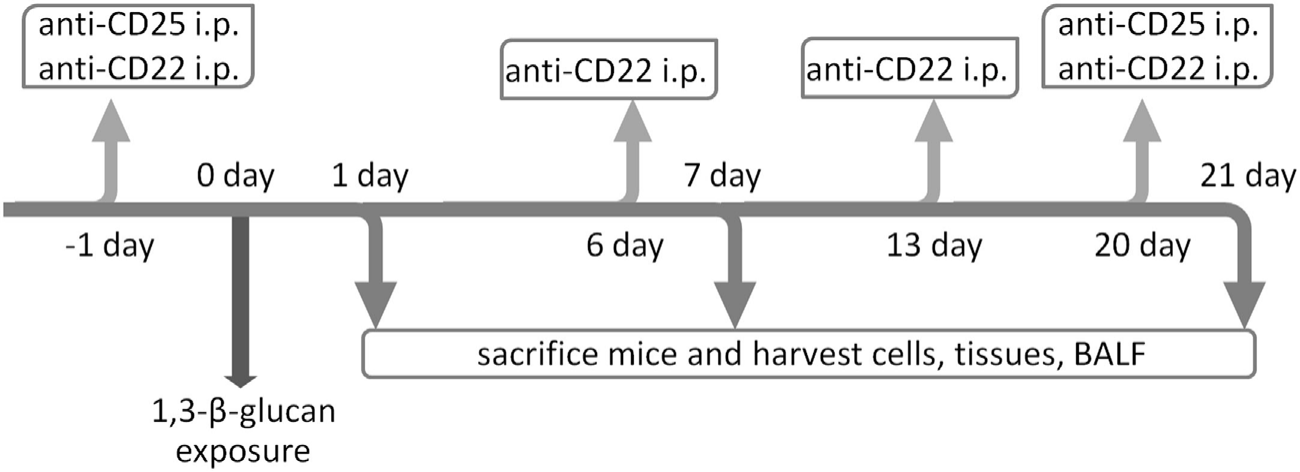
**Schematic diagrams of the experimental design**. To deplete IL-10-producing B cells, C57BL/6 mice were treated i.p. with anti-CD22 Ab on 1 day before 1,3-β-glucan exposure and on days 6, 13, and 20 after 1,3-β-glucan exposure for continuous depletion. Anti-CD25 Ab was applied on either 1 day before 1,3-β-glucan exposure or on day 20 after 1,3-β-glucan exposure to deplete regulatory T cell on the two separate stages during 1,3-β-glucan-induced lung inflammation. Mice were sacrificed on days 1, 7, and 21. The bronchoalveolar lavage fluid (BALF), tissues, and cells were collected for the following assay.

The whole experimental procedure was showed in Figure 1. In brief, mice were sacrificed on 1, 7, or 21 days after 1,3-β-glucan exposure. Bronchoalveolar lavage fluid (BALF) was obtained and centrifuged at 1,500 rpm for 8 min at 4°C. RBCs were lysed, and the BALF cell pellet was washed and resuspended in phosphate-buffered saline (PBS). The total cell counts were determined using standard hematological procedures. BALF cytospin was prepared and stained using the Wright–Giemsa method. In brief, three major inflammatory cells could be observed under the optical microscope (200×): polymorphonuclear neutrophils, small and medium-sized lymphocytes, and large macrophages with visible cytoplasm. Neutrophils, macrophages, and lymphocytes were identified in a population of 200 cells using standard morphological criteria.

### Pathological Examination

Mice lungs were fixed in 4% paraformaldehyde–PBS. The tissue was embedded in paraffin and cut into 6 μm thick sections. The tissue sections were stained with H&E for pathological examination. In general, slides were viewed under Olympus BX51 microscope, and photographic images of lung morphology were captured at 10 × 20 magnification. Lung inflammation was graded into four stages and scored as follows (33): 0 point—normal lung; 1 point—light inflammation, minimal inflammatory thickening of alveolar walls, limited to the local area involving <20% of lung area, and normal alveolar structure; 2 points—medium inflammation and involving 20–50% inflammation of the entire lung area; 3 points—heavy inflammation, lesion involving > 50% of the lung area, severe structure distortion, monocyte infiltration into the alveolar space, and presence of consolidation.

### Isolation of Hilar Lymph Nodes (HLNs) and Spleen Cells

Hilar lymph nodes were harvested, dissected with dissection needles, and digested with 0.25% trypsin at 37°C for 5 min. Then, 3% fetal bovine serum PBS was used to quench the digestion. Samples were centrifuged at 1,500 rpm for 8 min at 4°C. The HLN cell pellet was washed and resuspended in PBS. The spleen was removed, ground, and mechanically dissociated in cold PBS. After lysis of RBCs, spleen cells were washed and resuspended in PBS.

### Flow Cytometry

The HLN and spleen cells were stimulated with Leukocyte Activation Cocktail (BD Pharmingen, San Jose, CA, USA) and LPS 10 μg/ml (Sigma-Aldrich, St. Louis, MO, USA) for 5 h, followed by blocking with purified rat anti-mouse CD16/CD32 (2.4G2; BD Pharmingen) for 10 min at 4°C. Cell-surface staining was performed with PerCP-Cy5.5 conjugated CD4 (RM4-5; BD Pharmingen) or PerCP-Cy5.5 conjugated CD19 (1D3; BD Pharmingen) as described previously (9). Cells were fixed and permeabilized using a Fixation/Permeabilization kit (eBioscience, San Diego, CA, USA) or BD Cytofix/Cytoperm™ Fixation/Permeabilization Solution kit (BD Pharmingen) according to the manufacturer’s instructions. Then, cells were stained for 20 min at 4°C with Alexa Fluor 488-conjugated IFN-γ (XMG1.2; BD Pharmingen), PE-conjugated IL-17A (TC11-18H10; BD Pharmingen), Alexa Fluor 647-conjugated Foxp3 (MF23; BD Pharmingen), or PE-conjugated IL-10 (JES5-16E3; BD Pharmingen). Stained cells were washed twice in staining buffer (BD Pharmingen) and resuspended in 1% paraformaldehyde–PBS. Analysis of cell marker expression was performed using a FACSCanto II system (BD, Franklin Lakes, NJ, USA). Dead cells were gated out depending on forward scattering and side scattering. Cells were analyzed with FlowJo software.

### Cytomeric Bead Arrays (CBA)

Secreted protein levels in BALF were examined by CBA assay using mouse Th1/Th2/Th17 cytokine kit (BD Pharmingen) following the manufacturer’s instructions. Generally, multiple capture beads were mixed together, including TNF-α, IL-6, IFN-γ, IL-4, IL-17, and IL-10. The mixed capture beads were cocultured with 50 μl BALF supernatant and detection reagent for 2 h. The beads were washed carefully and suspended. Samples were analyzed by FACSCanto II system (BD). Data were analyzed with FCAP Array software.

### RNA Extraction and Real-time RT-PCR

Total RNA was isolated from lung homogenates using the TRIzol Reagent (Invitrogen, Carlsbad, CA, USA) according to the manufacturer’s instructions. The RNA concentration and the A260/A280 ratio were determined using a UV spectrophotometer.

Primers and Taqman probes were designed with Primer3,^1^ and the sequences were checked by performing a BLAST search.^2^ The primer sequences were as shown in Table 1. PrimeScript RT kit (DRR047A, Takara, Japan) and PrimeScript RT-PCR kit (DRR096A, Takara, Japan) were used for real-time RT-PCR analysis. The expressions of different cytokines in mice lung were determined using standard methodologies described previously (9).

**Table 1:** **The primer sequences used for real-time PCR**.

Target gene		Sequence
IL-6	Forward 5′–3′	CAACGATGATGCACTTGCAGA
	Reverse 5′–3′	CTCCAGGTAGCTATGGTACTCCAGA
TNF-α	Forward 5′–3′	ACTCCAGGCGGTGCCTATGT
	Reverse 5′–3′	GTGAGGGTCTGGGCCATAGAA
IFN-γ	Forward 5′–3′	AAGCGTCATTGAATCACACCTG
	Reverse 5′–3′	TGACCTCAAACTTGGCAATACTC
T-bet	Forward 5′–3′	TCAACCAGCACCAGACAGAGA
	Reverse 5′–3′	TCCACCAAGACCACATCCAC
IL-4	Forward 5′–3′	ACGGAGATGGATGTGCCAAAC
	Reverse 5′–3′	AGCACCTTGGAAGCCCTACAGA
IL-13	Forward 5′–3′	CCCCTGTGCAACGGCAGCAT
	Reverse 5′–3′	GAAGGGGCCGTGGCGAAACA
GATA3	Forward 5′–3′	GGATGTAAGTCGAGGCCCAAG
	Reverse 5′–3′	ATTGCAAAGGTAGTGCCCGGTA
IL-2	Forward 5′–3′	CCCAGGATGCTCACCTTCA
	Reverse 5′–3′	GCAGAGGTCCAAGTTCATCTTC
IL-17a	Forward 5′–3′	GCAAAAGTGAGCTCCAGAAGG
	Reverse 5′–3′	TCTTCATTGCGGTGGAGAGTC
IL-23	Forward 5′–3′	ACATGCACCAGCGGGACATA
	Reverse 5′–3′	CTTTGAAGATGTCAGAGTCAAGCAG
ROR-γt	Forward 5′–3′	ACGGCCCTGGTTCTCATCA
	Reverse 5′–3′	CCAAATTGTATTGCAGATGTTCCAC
Foxp3	Forward 5′–3′	CCCATCCCCAGGAGTCTTG
	Reverse 5′–3′	ACCATGACTAGGGGCACTGTA
CTLA-4	Forward 5′–3′	TCCGGAGGTACAAAGCTCAACTG
	Reverse 5′–3′	ACCATGGCTGCTAGCCAACAC
IL-10	Forward 5′–3′	GGGGCCAGTACAGCCGGGAA
	Reverse 5′–3′	CTGGCTGAAGGCAGTCCGCA
TGF-β	Forward 5′–3′	TGTGGAACTCTACCAGAAATATAGC
	Reverse 5′–3′	GAAAGCCCTGTATTCCGTCTC
GAPDH	Forward 5′–3′	CAATGTGTCCGTCGTGGATCT
	Reverse 5′–3′	GTCCTCAGTGTAGCCCAAGATG

### Statistical Analyses

Data were analyzed for statistical significance using SPSS 19.0 software. The differences between values were evaluated through a one-way analysis of variance followed by pair-wise comparison with the Student–Newman–Keuls test. *P* < 0.05 was considered statistically significant. All values are means ± SEM.

## Results

### B10 Participated in Controlling 1,3-β-Glucan-Induced Lung Inflammation

**Figure 2:**
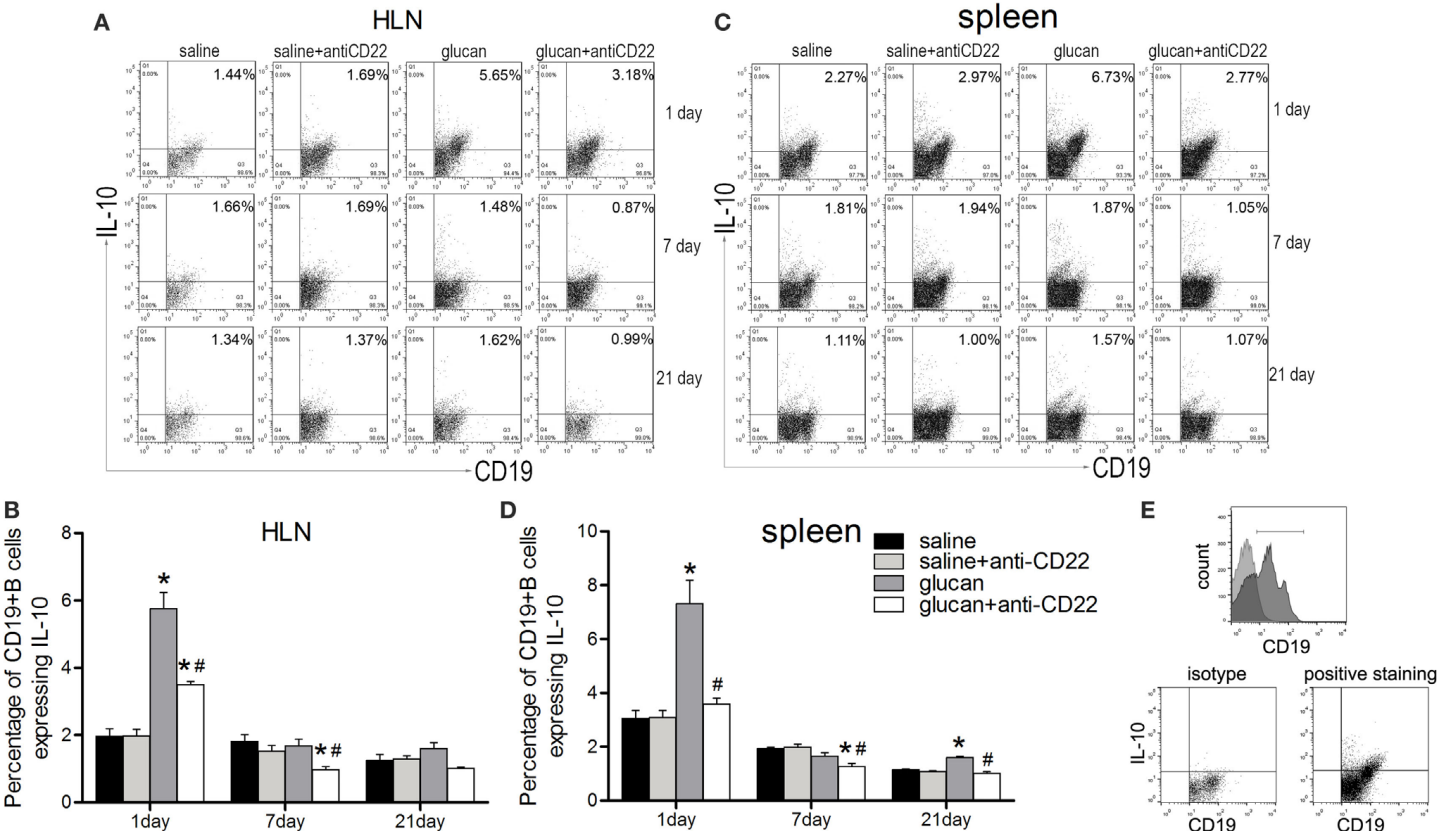
**Anti-CD22 treatment attenuates IL-10-producing B cells (B10) induction *in vivo* after 1,3-β-glucan exposure**. **(A,B)** C57BL/6 mice were treated i.p. with 300 μg anti-CD22 monoclonal antibody or control IgG, and the percentage of CD19^+^IL-10^+^ regulatory B cells (B10) in the hilar lymph node was assayed by flow cytometry. **(C,D)** Percentage of B10 in spleen is shown in the graph. **(E)** The gating strategy of B10 in flow cytometry (*n* = 5; *P < 0.05 compared with the saline group; ^#^*P* < 0.05 compared with the glucan group).

To study the role of B10 in 1,3-β-glucan-induced lung inflammation, B10 deficiency mice model was setup by injection of anti-CD22 antibody every 7 days. CD22 was dominantly expressed in B cells and was involved in the survival and regulation of B10 cells. Application of anti-CD22 antibody could preferentially deplete B10 in mice (34). In the present study, flow cytometry was performed to show whether B10 was involved in 1,3-β-glucan-induced lung inflammation. The percentage of CD19^+^IL-10^+^ B cells in the HLN increased obviously on day 1 after 1,3-β-glucan exposure (Figures 2A,B). Then, the percentage of CD19^+^IL-10^+^ B cell decreased to the same level as that in saline group mice at day 7 and day 21. Similar result was observed in spleen (Figures 2C,D). The isotype staining control was showed in Figure 2E. Next, to demonstrate its role, a B10 depletion mouse model was generated by anti-CD22 antibody injection. Compared with the glucan group, mice treated with anti-CD22 showed decreased percentage of CD19^+^IL-10^+^ B cell in both HLN and spleen on days 1, 7, and 21 after 1,3-β-glucan exposure. Meanwhile, anti-CD22 treatment could not influence the percentage of CD19^+^IL-10^+^ B cell in normal saline control mice. Therefore, anti-CD22 treatment could abrogate 1,3-β-glucan-induced B10 induction.

**Figure 3:**
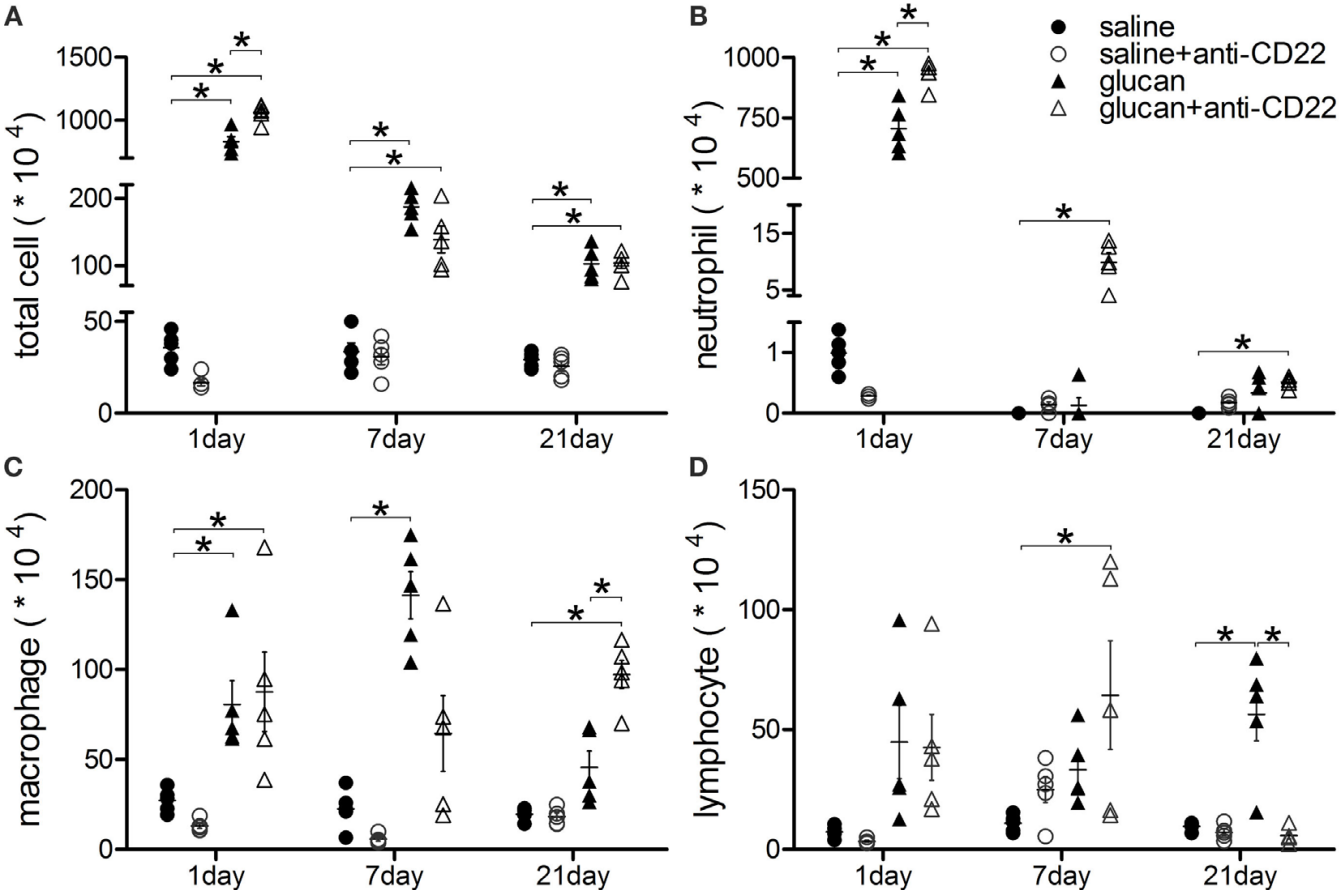
**Insufficient IL-10-producing B cells enhances accumulation of inflammatory cells in lung after 1,3-β-glucan exposure**. **(A)** Total cells, **(B)** neutrophils, **(C)** macrophages, and **(D)** lymphocytes in bronchoalveolar lavage fluid were counted using Giemsa staining. The experiment was repeated twice (*n* = 5; *P < 0.05 compared between groups).

The accumulation of inflammatory cells was observed after 1,3-β-glucan exposure. Giemsa–Wright staining was performed to count inflammatory cells in BALF. As showed in Figure 3, massive inflammatory cells accumulated on day 1 after 1,3-β-glucan exposure, including neutrophils, macrophages, and lymphocytes. Anti-CD22 treatment further increased the total inflammatory cells number in BALF on day 1 after 1,3-β-glucan exposure (Figure 3A). Although no significant difference of total cells was observed between the glucan group mice and the glucan + anti-CD22 group mice, anti-CD22 treatment induced an apparent increase of neutrophils and a considerable increase of lymphocytes on day 7 after 1,3-β-glucan exposure (Figures 3B,D). On day 21, anti-CD22 treatment significantly increased the accumulation of macrophages in BALF compared with that in the glucan group mice and the saline group mice (Figure 3C). Thus, anti-CD22 treatment led to the accumulation of more inflammatory cells in BALF, primarily early neutrophils, lymphocytes, and late macrophages after 1,3-β-glucan exposure.

**Figure 4:**
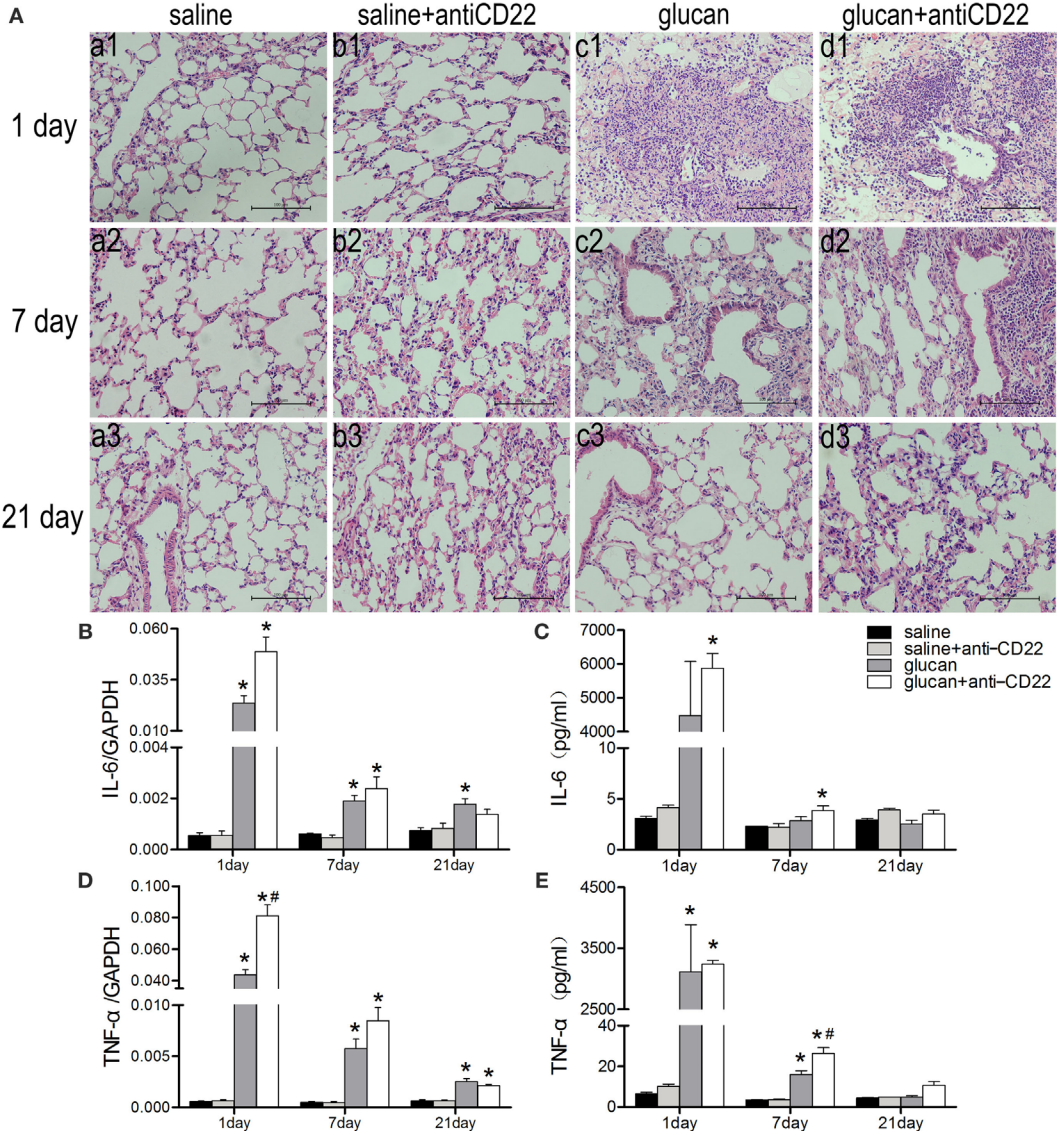
**Insufficient IL-10-producing B cells exacerbates 1,3-β-glucan-induced inflammatory response**. **(A)** Histopathology changes in mouse lungs after silica instillation observed with H&E staining (×200). a1–d1, day 1; a2–d2, day 7, and a3–d3, day 21. a1–a3, saline group; b1–b3, saline + anti-CD22 group; c1–c3, glucan group; d1–d3, glucan + anti-CD22 group. Secretions of IL-6 **(C)** and TNF-α **(E)** in bronchoalveolar lavage fluid were evaluated by cytomeric bead arrays analysis (*n* = 3). Expressions of IL-6 **(B)** and TNF-α **(D)** in lung were assayed by real-time PCR (*n* = 5–6; *P < 0.05 compared with the saline group; ^#^*P* < 0.05 compared with the glucan group).

To study the impact of B10 on the pathogenesis of 1,3-β-glucan-induced lung inflammation, the pathological changes of mice lung were observed after H&E staining. And the pathological score of lung inflammation was showed in Table 2. No obvious abnormalities were observed in the saline group and the saline + anti-CD22 group, which indicated that anti-CD22 treatment could not induce obvious pathological changes in lung (Figure 4A). Mice in the glucan group showed a large infiltration of inflammatory cells and exhibited alveolar septal thickening on day 1 after 1,3-β-glucan exposure. And then, a considerably reduced inflammation was observed on day 7 after 1,3-β-glucan exposure. The inflammation in the glucan group mice almost recovered on day 21 after 1,3-β-glucan exposure. Compared with the glucan group, mice in the glucan + anti-CD22 group demonstrated severer infiltration of inflammatory cells on day 1. Mice treated with anti-CD22 continuously showed the infiltration of inflammatory cell and thickened alveolar septal on day 7 after 1,3-β-glucan exposure. The remission of inflammation was not observed in the glucan + anti-CD22 group even on day 21 after 1,3-β-glucan exposure. Taken together, anti-CD22 treatment exacerbated the inflammatory pathology changes in mice lung after 1,3-β-glucan exposure.

**Table 2:** **Inflammation score in each group at days 1, 7, and 21**.

Groups	1 day after 1,3-β-glucan exposure	7 days after 1,3-β-glucan exposure	21 days after 1,3-β-glucan exposure
Saline	0.060 ± 0.040	0.020 ± 0.013	0.030 ± 0.020
Saline + anti-CD22	0.04 ± 0.024	0.024 ± 0.019	0.028 ± 0.017
Glucan	2.120 ± 0.086*	1.520 ± 0.171*	0.920 ± 0.128*
Glucan + anti-CD22	2.840 ± 0.060*,#	2.300 ± 0.071*,#	1.720 ± 0.066*,#

The degree of inflammation was assessed by histological analysis of six random fields per sample (*n* = 5; *P < 0.05 compared with the saline group; ^#^*P* < 0.05 compared with the glucan group).

To further evaluate the inflammatory changes in lung, the representative pro-inflammatory cytokines, IL-6 and TNF-α, were examined by real-time PCR and CBA. 1,3-β-Glucan exposure led to increased expressions and secretions of both IL-6 and TNF-α in mice lung and BALF on day 1. The expression of TNF-α in the glucan + anti-CD22 group was increased significantly compared with that in glucan group on day 1 (Figure 4D). The secretion of TNF-α in the glucan + anti-CD22 group was increased significantly on day 7 (Figure 4E). The expression and secretion of IL-6 also got a considerable increase in glucan + anti-CD22 group mice compared with glucan group mice on day 1 (Figures 4B,C). The enhanced levels of IL-6 and TNF-α induced by anti-CD22 treatment were still obvious even on day 7 after 1,3-β-glucan exposure compared with the glucan group. These combined results indicate that insufficient B10 could aggravate the lung inflammation after 1,3-β-glucan exposure.

### B10 Regulated Th Immune Responses during 1,3-β-Glucan-Induced Lung Inflammation

According to existing studies, Th immune responses played crucial roles in the development of 1,3-β-glucan-induced lung inflammation. Th1 response was elevated at early stage, and Th2 response became predominant at late stage. In addition, Th17 response also took part in the inflammatory progress. To study whether B10 regulated 1,3-β-glucan-induced lung inflammation by affecting Th immune responses, the phenotype of CD4^+^ T lymphocyte in HLN and the levels of cytokines were checked.

**Figure 5:**
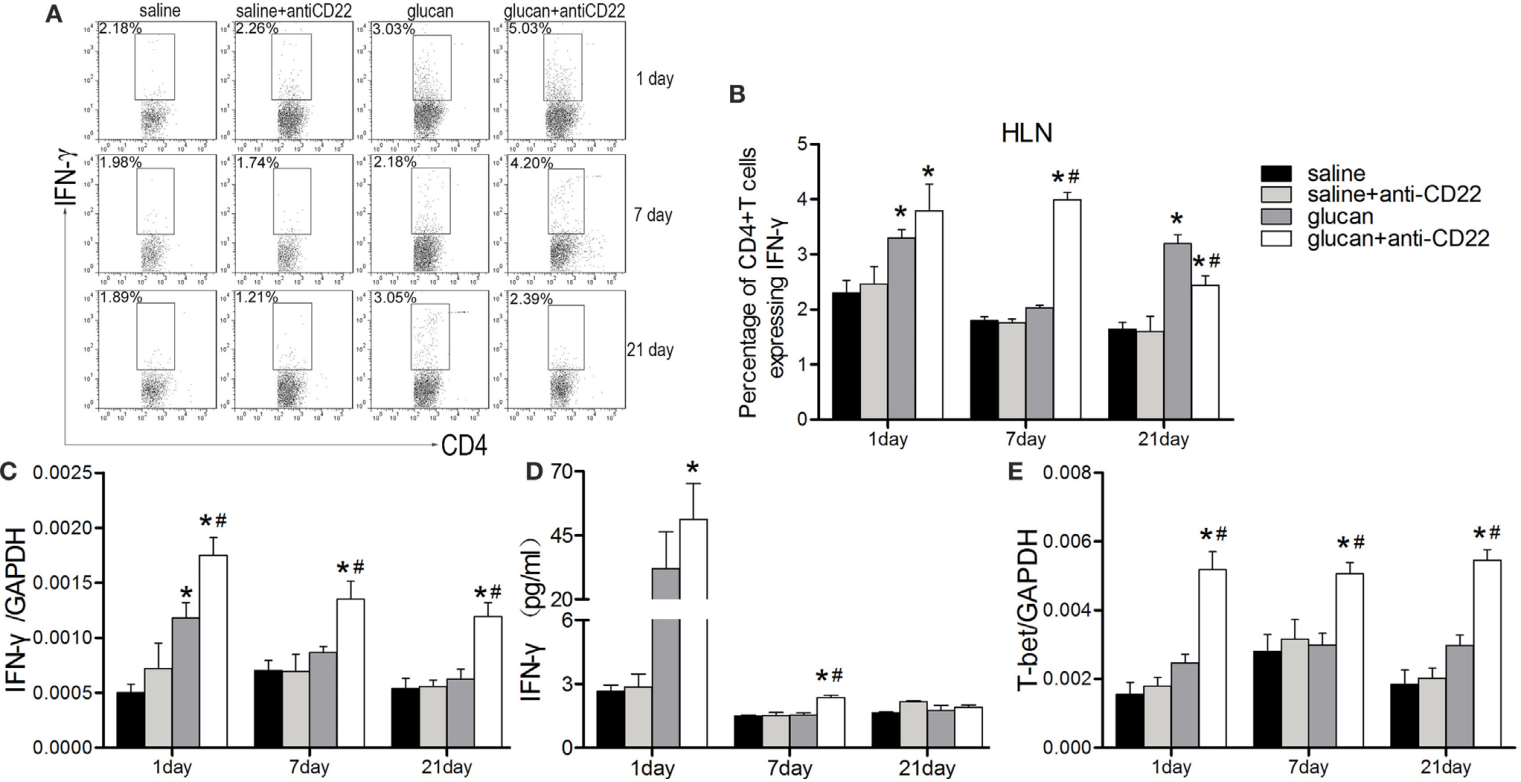
**Insufficient IL-10-producing B cells promotes the Th1 response during 1,3-β-glucan-induced lung inflammation**. **(A,B)** Percentage of CD4^+^IFN-γ^+^ Th1 cells in the hilar lymph node was assayed by flow cytometry. Secretion of typical Th1 cytokine IFN-γ **(D)** was assayed by cytomeric bead arrays (*n* = 3). Expression of typical Th1 cytokine IFN-γ **(C)** and its typical transcription factor T-bet **(E)** were assayed by real-time PCR (*n* = 5–6; *P < 0.05 compared with the saline group; ^#^*P* < 0.05 compared with the glucan group).

As showed in Figure 5, the percentage of IFN-γ-producing CD4^+^ T cell (Th1 cell) was increased in anti-CD22-treated mice at early stage (days 1 and 7) after 1,3-β-glucan exposure (Figures 5A,B). Anti-CD22 treatment increased the level of typical Th1 cytokine, IFN-γ, on day 1 after 1,3-β-glucan exposure. And the increase of IFN-γ in the glucan + anti-CD22 group was still obvious on day 7 while its level in the glucan group reduced to the control level (Figures 5C,D). Although its expression could be associated with many immune cells, T-bet was originally considered as the typical Th1 transcription factor, which plays important roles in coordinating immune response. In this study, the expression of T-bet was increased considerably by anti-CD22 treatment after 1,3-β-glucan exposure (Figure 5E). These data suggest that insufficient B10 enhanced the Th1 response at early stage of lung inflammation induced by 1,3-β-glucan.

**Figure 6:**
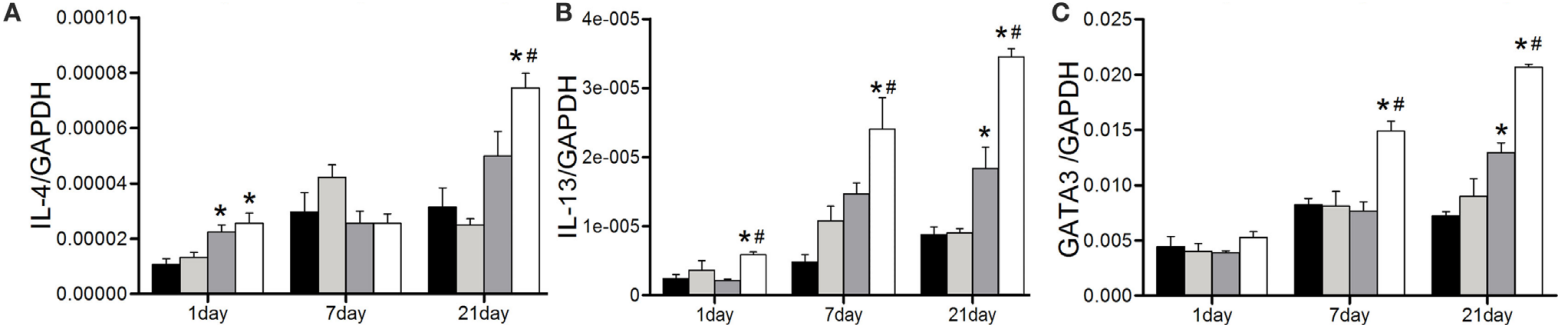
**Insufficient IL-10-producing B cells increases the Th2 response at the late stage of 1,3-β-glucan-induced lung inflammation**. Expressions of typical Th2 cytokine IL-4 **(A)**, IL-13 **(B)**, and its typical transcription factor GATA3 **(C)** were assayed by real-time PCR (*n* = 5–6; *P < 0.05 compared with the saline group; ^#^*P* < 0.05 compared with the glucan group).

Next, the expressions of Th2 representative cytokines were examined by real-time PCR. In line with our previous study, the elevated IL-4 expression was observed at the late stage after 1,3-β-glucan exposure. Anti-CD22 treatment apparently increased its expression on day 21 compared with that in the glucan group (Figure 6A). Similar tendency was observed in the expression of IL-13. The obvious IL-13 induction did not appeared until day 21 after 1,3-β-glucan exposure. However, anti-CD22 treatment increased the expression of IL-13 earlier on day 1 after 1,3-β-glucan exposure and kept its level continuously rising up to peak on day 21 (Figure 6B). The expression of typical Th2 transcription factor GATA3 increased significantly in the glucan + anti-CD22 group mice compared with that in the glucan group (Figure 6C). This indicates that insufficient B10 elevated Th2 response at late stage of 1,3-β-glucan-induced lung inflammation.

**Figure 7:**
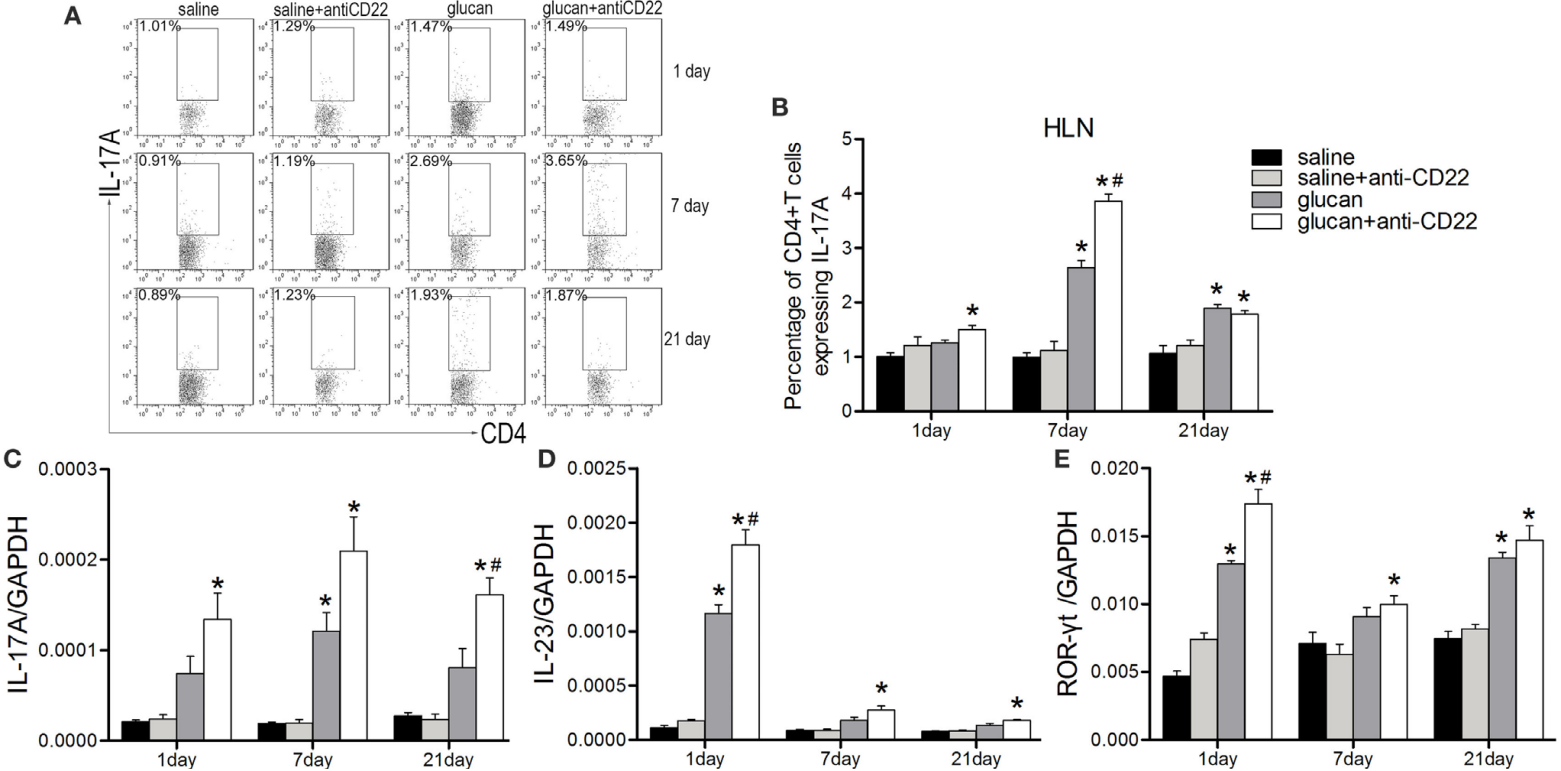
**Insufficient IL-10-producing B cells increases the Th17 response during 1,3-β-glucan-induced lung inflammation**. Percentage of CD4^+^IL-17A^+^ Th17 cells in the hilar lymph node was assayed by flow cytometry **(A,B)**. Expressions of typical Th17 cytokine IL-17A **(C)**, IL-23 **(D)**, and its typical transcription factor ROR-γt **(E)** were assayed by real-time PCR (*n* = 5–6; *P < 0.05 compared with the saline group; ^#^*P* < 0.05 compared with the glucan group).

Besides, Th17 response also was studied when mice were treated with anti-CD22 (Figure 7). Flow cytometry results demonstrated that the percentage of IL-17-producing CD4^+^ T cell (Th17 cell) was higher obviously in the glucan + anti-CD22 group than that in the glucan group especially on day 7 after 1,3-β-glucan exposure (Figures 7A,B). The expressions of representative Th17 cytokines also confirmed the role of anti-CD22 treatment. The levels of both IL-17A and IL-23 were clearly higher in the glucan + anti-CD22 group than that in the glucan group on day 1, so was the expression of ROR-γt, typical Th17 transcription factor (Figures 7C–E). These data suggest that insufficient B10 also promoted Th17 response in 1,3-β-glucan-induced lung inflammation.

### The Dual Relationship between B10 and Treg in Regulating 1,3-β-Glucan-Induced Lung Inflammation

**Figure 8:**
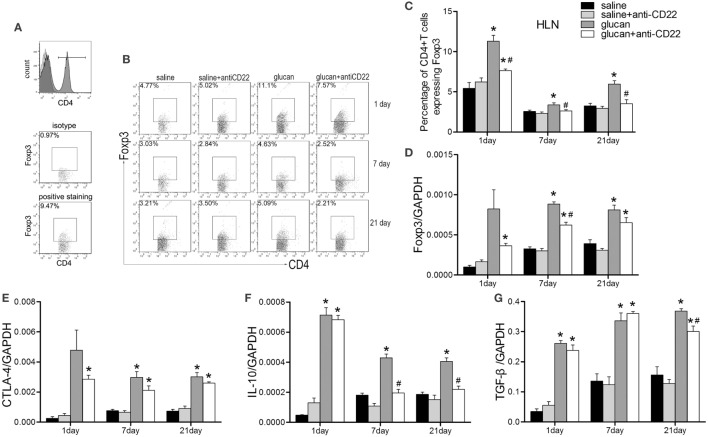
**Insufficient IL-10-producing B cells restricts the regulatory T cell (Treg) response during 1,3-β-glucan-induced lung inflammation**. **(A)** The gating strategy of Treg in flow cytometry. Percentage of CD4^+^Foxp3^+^ Treg cells in the hilar lymph node was assayed by flow cytometry **(B,C)**. Expressions of Treg functional molecules Foxp3 **(D)**, CTLA-4 **(E)**, inhibitory cytokines IL-10 **(F)**, and TGF-β **(G)** were assayed by real-time PCR (*n* = 5–6; *P < 0.05 compared with the saline group; ^#^*P* < 0.05 compared with the glucan group).

According to our previous studies, Treg was vital for the regulation of Th immune response during 1,3-β-glucan-induced lung inflammation. Here, we evaluated whether Treg was involved in B10 regulation of Th responses. Results from flow cytometry demonstrated that anti-CD22-treated mice showed lower percentage of Treg after 1,3-β-glucan exposure than glucan group mice based on its isotype staining control (Figures 8A–C). And the expression of foxp3, typical Treg transcription factor, decreased obviously in the glucan + anti-CD22 group mice compared with that in the glucan group (Figure 8D). What’s more anti-CD22 treatment not only reduced the number of Treg but also affected its functional factor CTLA-4. Real-time PCR discovered considerable restricted level of CTLA-4 in anti-CD22-treated mice on day 1 compared with mice in the glucan group (Figure 8E). When 1,3-β-glucan-induced lung inflammation developed into the late stage, anti-CD22 treatment significantly limited the increase of IL-10 compared with that in glucan group (Figure 8F). Besides, TGF-β expression also exhibited lower level in anti-CD22-treated mice than that in the glucan group (Figure 8G). These data suggest that insufficient B10 could limit Treg and influence IL-10 during 1,3-β-glucan-induced lung inflammation.

**Figure 9:**
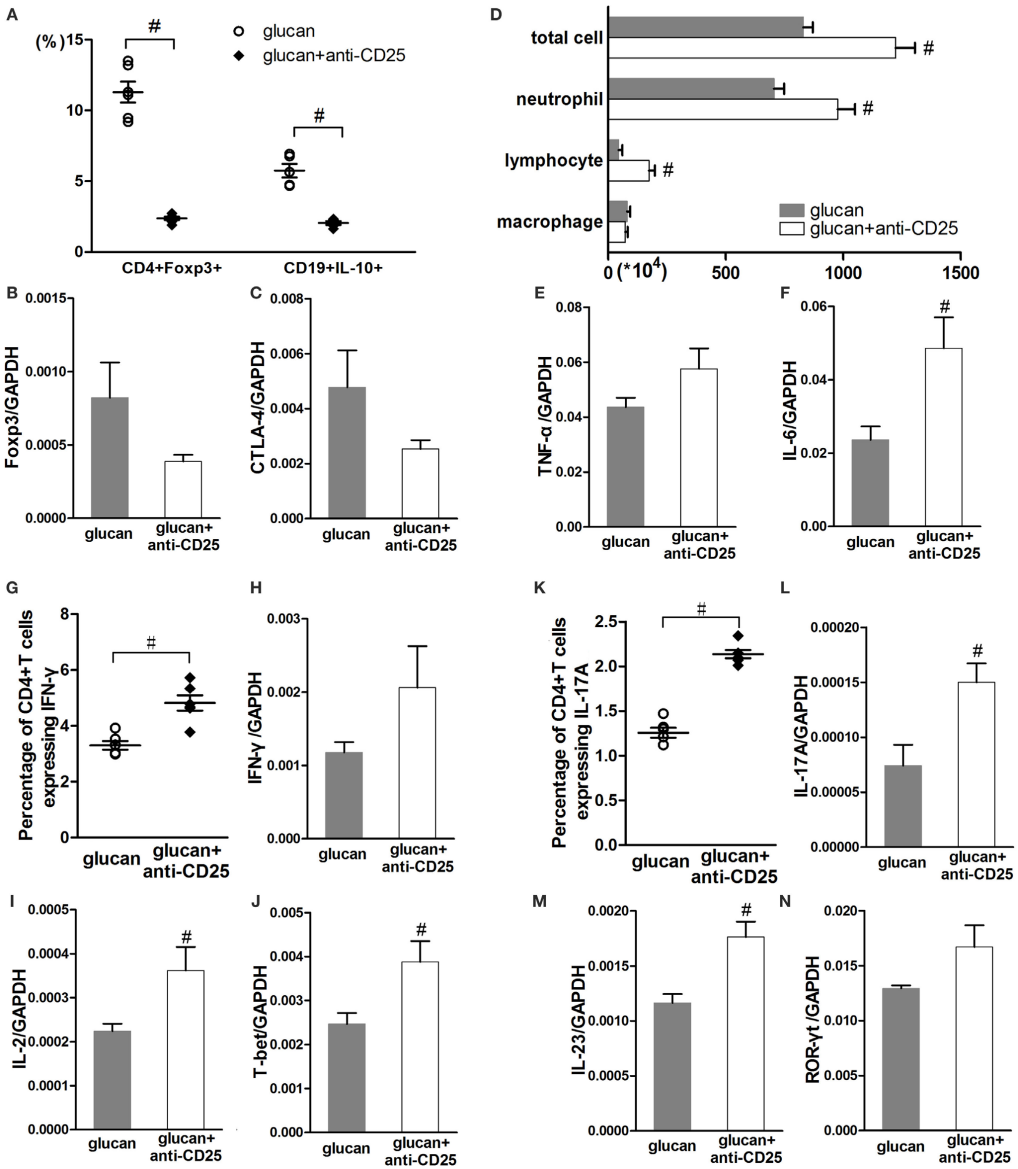
**Regulatory T cell (Treg) depletion at the early stage after 1,3-β-glucan exposure could limit IL-10-producing B cells (B10) and promotes inflammatory responses**. Percentages of Treg cells and B10 in the hilar lymph node were assayed by flow cytometry **(A)**. Expressions of Treg functional molecules Foxp3 **(B)** and CTLA-4 **(C)** were assayed by real-time PCR. Total cells, neutrophils, macrophages, and lymphocytes **(D)** in bronchoalveolar lavage fluid were counted using Giemsa staining. Expressions of TNF-α **(E)** and IL-6 **(F)** in lung were assayed by real-time PCR. Th1 response, including the percentage of Th1 cells **(G)**, the expressions of IFN-γ **(H)**, IL-2 **(I)**, and T-bet **(J)** was checked by flow cytometry and real-time PCR. Th17 response, including the percentage of Th17 cells **(K)**, the expressions of IL-17A **(L)**, IL-23 **(M)**, and ROR-γt **(N)** was checked by flow cytometry and real-time PCR (*n* = 5–6; ^#^*P* < 0.05 compared with the glucan group).

Considering the controversial relationship between Treg and B10 based on existing studies, anti-CD25 antibody was used to deplete Treg in mice at early or late stage separately. As showed in Figure 9, anti-CD25 injection depleted Treg successfully and reduced its functional factors, Foxp3 and CTLA-4 (Figures 9A–C). Treg depletion led to obviously reduced percentage of B10 in mice HLN (Figure 9A). Like changes caused by insufficient B10, Treg depletion led to increased infiltration of inflammatory cells, such as neutrophils and lymphocytes on day 1 after 1,3-β-glucan exposure (Figure 9D). The expressions of pro-inflammatory cytokines, TNF-α and IL-6, increased to a variable extent after Treg depletion (Figures 9E,F). On the one hand, Treg-depleted mice exhibited higher percentage of Th1 cell on day 1 than mice in the glucan group (Figure 9G). The expressions of IL-2 and T-bet were also elevated by Treg depletion (Figures 9I,J). On the other hand, the percentage of Th17 cell increased significantly in Treg-depleted mice (Figure 9K). Levels of classical cytokines and transcript factor of Th17 response, IL-17A, IL-23, and ROR-γt ascended a lot by Treg depletion (Figures 9L–N). These data show that depleting Treg at the early stage could restrict 1,3-β-glucan-induced B10 induction, which indicates a reciprocity between B10 and Treg.

**Figure 10:**
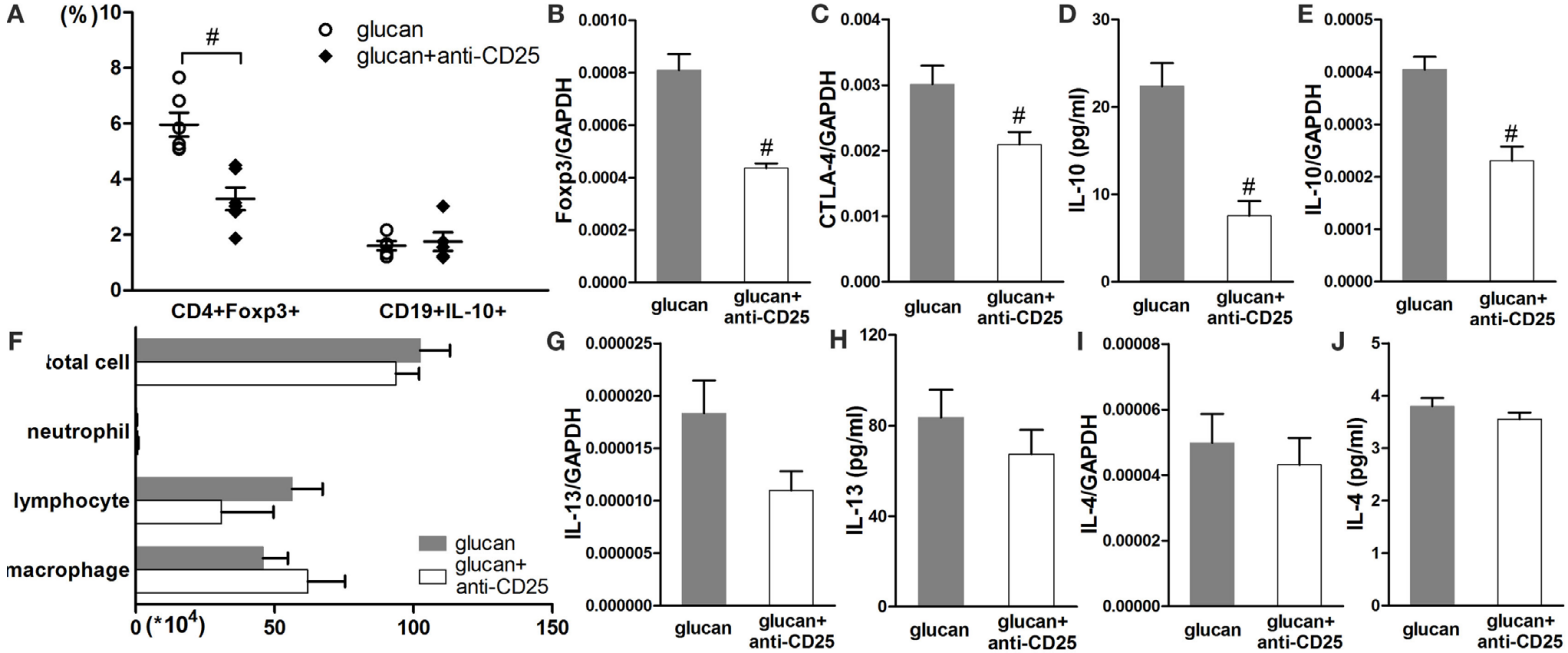
**Regulatory T cell (Treg) depletion at the late stage after 1,3-β-glucan exposure could not affect IL-10-producing B cells (B10)**. Percentages of Treg cells and B10 in the hilar lymph node were assayed by flow cytometry **(A)**. Expressions of Treg functional molecules Foxp3 **(B)**, CTLA-4 **(C)**, and inhibitory cytokine IL-10 **(E)** were assayed by real-time PCR. Secretion of inhibitory cytokine IL-10 **(D)** in BAL was assayed by cytomeric bead arrays. Total cells, neutrophils, macrophages, and lymphocytes **(F)** in bronchoalveolar lavage fluid were counted using Giemsa staining. Expressions of typical Th2 cytokine IL-13 **(G)** and IL-4 **(I)** were check by real-time PCR. Secretions of typical Th2 cytokine IL-13 **(H)** and IL-4 **(J)** were check by real-time PCR (*n* = 5–6; ^#^*P* < 0.05 compared with the glucan group).

At late stage of 1,3-β-glucan-induced lung inflammation, Treg-depleted mice exhibited differently from mice with insufficient B10. As showed in Figure 10, anti-CD25 injection successfully reduced the percentage of Treg and lowered the expressions of Foxp3 and CTLA-4 on day 21 after 1,3-β-glucan exposure (Figures 10A–C). However, Treg depletion did not influence the percentage of B10 (Figure 10A). There was no obvious difference in inflammatory cell accumulation between Treg-depleted mice and mice in the glucan group on day 21 after 1,3-β-glucan exposure (Figure 10F). In addition, the expression and secretion of IL-13 decreased apparently by Treg depletion at late stage of lung inflammation (Figures 10G,H). Although B10 was not influenced by Treg depletion, the level of IL-10 decreased significantly in mice with anti-CD25 injection compared with that in the glucan group (Figures 10D,E). These data indicate that depleting Treg at late stage of 1,3-β-glucan-induced lung inflammation was not able to affect B10.

## Discussion

The regulatory role of B cells was not identified until recently (35). Studies showed that B cells participated in regulating several T cell-associated diseases, such as inflammation, autoimmune disease, and tumorigenesis (36). And their diverse phenotypes were associated with CD1d, CD5, TIM-1, CD38, and CD24 cell-surface markers in mice and humans. However, none of these markers uniquely defined all IL-10-producing B cells (B10), which were reported to participate in chronic inflammation and allergic disease (14, 27, 37). Thus, CD19 and IL-10 were used to delineate B10 in the present study. Previous studies on regulatory B cells used CD19 knockout mice, anti-CD20 antibody neutralization, or IL-10 knockout mice (38). However, these methods influenced other B cell subsets or other IL-10-producing cells in addition to B10. CD22 was called inhibitory BCR co-receptors (39). Although some of other BCR co-receptors were expressed in other immune cell types such as dendritic cells, CD22 was dominantly expressed on B cells (40). Study demonstrated that CD22 could regulate B cell through both ligand-dependent and ligand-independent way (41). CD22 was considered to play an important role in regulating B cells by binding to its ligand (42). Animal study confirmed that anti-CD22 antibody could preferentially deplete B10 in mice (34). It’s reasonable that CD22 engagement is particularly essential for B10 cells. Thus, anti-CD22 antibody was used in the current study to generate a B10 deficiency mouse model. Flow cytometry showed that anti-CD22 treatment limited B10 induction in mice after 1,3-β-glucan exposure. No obvious difference was observed between B10 percentage in saline group and B10 percentage in saline + anti-CD22 group. It’s reasonable to assume that anti-CD22 treatment contributed to deplete the inducible B10.

IL-10-producing B cells (B10) have been characterized as a suppressive immune regulatory cell. Lack of B10 could amplify the allergic inflammation (43). In the present study, inadequate endogenous B10 led to intensive accumulation of inflammatory cells, including neutrophils, lymphocytes, and macrophages, which was similar to that in OVA-immunized mice (22). The excessive inflammatory cells secreted overmuch pro-inflammatory cytokines, such as TNF-α and IL-6, which could further recruit much more inflammatory cells. Although TNF-α and IL-6 could be secreted by a variety of cells, such as activated macrophages, neutrophils, natural killer cells, T cells, B cells, fibroblast, etc. In this study, neutrophils, macrophages, and lymphocytes were three major contributors to the increase of TNF-α and IL-6. Since the most obvious changes of TNF-α and IL-6 were observed at day 1, when the changes of total cells were changed a lot. And the increase of TNF-α and IL-6 was not so significant when the changes of total cells showed no difference at day 7 and day 21. However, besides these three types, epithelial cells were also considered to be a source of these two pro-inflammatory cytokines. Insufficient B10 aggravated the inflammation, which was accompanied by the appearance of exacerbated inflammatory cells accumulation, prolonged inflammatory response, continual thickened alveoli septum, and severe destroyed alveolar structure. According to our dynamic observation on pathological changes, insufficient B10 influenced both the initiation and the development of lung inflammation. The suppressive function of B10 in lung inflammation was also testified in other inflammatory diseases, such as asthma and enteritis (34).

Although the detail mechanism of how it participated in T cell immune responses is not clear, regulatory B cells were clarified to be associated with Th immune responses, either effector T cell responses or Treg response (36). In the current study, B10 exerted its regulatory function *via* inhibiting Th1/Th17 responses in early stage and suppressing Th2 response in late stage. Our study demonstrated that insufficient B10 amplified the Th1 response, according to the increased number of Th1 cells, elevated levels of Th1 cytokine IFN-γ, and the continued high level of Th1 typical transcription factor T-bet. And this outcome was in accord with other studies (25, 44). On the other hand, T-bet is reported to be expressed in many cells of the adaptive and innate immune system, such as natural killer cells and dendritic cells, which might contribute to its continued high level at late time points. Transferring B10 to IL-10 knockout mice inhibited Th1 response in arthritis (45). The proliferation of Th1 cells was not affected by B10 yet *in vitro*. Insufficient B10 played an uncertain role in affecting Th17 response. In the current study, anti-CD22 treatment led to higher level of Th17 response. The negative correlation between B10 and Th17 response was also reported (46). Others believed that B10 could inhibit Th17 response only when Th1 response was absent (47). However, the role of B10 in influencing Th2 response is still debatable. Some studies believed that B10 could promote Th2 response, according to its regulation on Th1/Th2 balance. On the contrary, others illustrated that B10 could control airway disease in Th2-predominant asthma mice (17). In our study, insufficient B10 increased the levels of Th2 cytokines, IL-4 and IL-13. Insufficient B10 also increased Th2 transcription factor GATA3, which might elevate the expressions of Th2 cytokines feedback. Taken together, B10 could suppress Th1, Th2, and Th17 responses to varying extent in different stage after 1,3-β-glucan exposure.

Previous studies already demonstrated that Treg could obviously regulate 1,3-β-glucan-induced lung inflammation (9, 10). Similarly, as a regulatory immune cell subset, whether the regulation of B10 was associated with Treg? The present study showed that both B10 and Treg were significantly increased in lung inflammation, which indicated that B10 may also be involved in the induction of Treg after 1,3-β-glucan exposure. The increased B10 and Treg contributed to regulate the 1,3-β-glucan-induced lung inflammation as a result. However, not like the situation of saline group mice, B10 deficiency mice showed the overwhelming inflammation after 1,3-β-glucan exposure, which could not be controlled. It’s reasonable to hypothesize that Treg might be affected by two forces, inflammatory response and the number of B10. It seems that in this study, the influence of B10 was dominant, which may need further study to complete testified. Next, what’s the relationship between B10 and Treg? Some studies indicated that B10 could increase Treg accumulation through converting resting non-Treg into Treg (48, 49). Others believed that Treg could act independently and was not affected by B cell depletion (50). According to the current study, insufficient B10 could reduce the number of Treg, as well as the expressions of its typical transcription factor Foxp3 and its functional molecular CTLA-4. Our study indicates that B10 exerted its suppressive function through promoting Treg response in 1,3-β-glucan-induced lung inflammation. Besides of Treg, B10 regulation was based on either cell–cell contact through surface molecules or by releasing cytokines (51). Insufficient B10 affected the levels of inhibitory cytokine IL-10 and TGF-β during the late stage of 1,3-β-glucan-induced lung inflammation, which suggested that regulatory function of B10 was associated with inhibitory cytokine IL-10.

**Figure 11:**
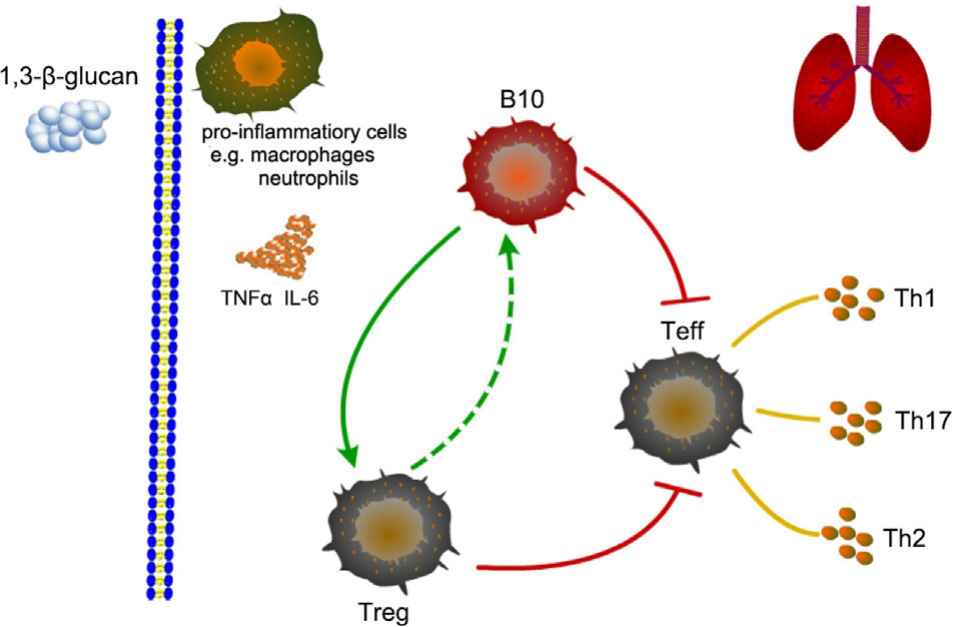
**Schematic representation of the role of B10 in modulating T helper (Th) immune responses after 1,3-β-glucan exposure in mice lung**.

In the opposite direction, whether Treg could also affected B10? Our study showed that the immune regulation caused by early Treg depletion was almost same as the effect resulted from insufficient B10. And depleting Treg before 1,3-β-glucan exposure decreased the number of B10. This might because that Treg could help B10 activation through TLRs ligation (20). B10 and Treg homeostasis need interacted with each other (52). Whereas, depleting Treg at late stage after 1,3-β-glucan exposure was not capable to affect the number of B10. And its modulation on immune response was different from that caused by insufficient B10. This might attribute to that Treg depletion occurred after B10 activation, when B10 could be maintained by other cells or molecules (36). Thus, it could be seen that there was a dual relationship between B10 and Treg. These two regulatory subsets could coordinate with each other at the early stage of 1,3-β-glucan-induced lung inflammation. However, at the late stage, the regulatory role of B10 was independent with Treg. As showed in Figure 11, our results indicated that B10 and the relationship between B10 and Treg might have potential from the perspective of inflammation treatment.

## Ethics Statement

This study was carried out in accordance with the recommendations of the National Institute of Health Guide for the Care and Use of Laboratory Animals and Animal Care and Use Committee at the China Medical University. The protocol was approved by the Animal Care and Use Committee at the China Medical University (CMU62043022).

## Author Contributions

FL and JC conceived and designed the research; FL, XL, WD, CL, SD, and YL performed experiments; FL, XL, and WD analyzed data; FL and DW interpreted results; FL prepared figures and drafted the manuscript; WD, YC, DW, and JC edited and revised the manuscript; all authors approved the final manuscript version.

## Conflict of Interest Statement

The authors declare that the research was conducted in the absence of any commercial or financial relationships that could be construed as a potential conflict of interest.

## Footnotes

^1^http://bioinfo.ut.ee/primer3-0.4.0/primer3/.

^2^http://blast.ncbi.nlm.nih.gov/Blast.cgi.
